# Structural Perturbations to Population Skeletons: Transient Dynamics, Coexistence of Attractors and the Rarity of Chaos

**DOI:** 10.1371/journal.pone.0024200

**Published:** 2011-09-19

**Authors:** Brajendra K. Singh, Paul E. Parham, Chin-Kun Hu

**Affiliations:** 1 Institute of Physics, Academia Sinica, Nankang, Taipei, Taiwan, Republic of China; 2 Interdisciplinary Centre for Human and Avian Influenza Research, Centre for Infectious Diseases, University of Edinburgh, Edinburgh, United Kingdom; 3 Department of Infectious Disease Epidemiology, Grantham Institute for Climate Change, Imperial College London, London, United Kingdom; University of Maribor, Slovenia

## Abstract

**Background:**

Simple models of insect populations with non-overlapping generations have been instrumental in understanding the mechanisms behind population cycles, including wild (chaotic) fluctuations. The presence of deterministic chaos in natural populations, however, has never been unequivocally accepted. Recently, it has been proposed that the application of chaos control theory can be useful in unravelling the complexity observed in real population data. This approach is based on structural perturbations to simple population models (population skeletons). The mechanism behind such perturbations to control chaotic dynamics thus far is model dependent and constant (in size and direction) through time. In addition, the outcome of such structurally perturbed models is [almost] always equilibrium type, which fails to commensurate with the patterns observed in population data.

**Methodology/Principal Findings:**

We present a proportional feedback mechanism that is independent of model formulation and capable of perturbing population skeletons in an evolutionary way, as opposed to requiring constant feedbacks. We observe the same repertoire of patterns, from equilibrium states to non-chaotic aperiodic oscillations to chaotic behaviour, across different population models, in agreement with observations in real population data. Model outputs also indicate the existence of multiple attractors in some parameter regimes and this coexistence is found to depend on initial population densities or the duration of transient dynamics. Our results suggest that such a feedback mechanism may enable a better understanding of the regulatory processes in natural populations.

## Introduction

A central theme in population ecology is to understand the mechanisms of survival and extinction in animal populations [1]–[5]. Simple mathematical models of insect populations where generations do not overlap have served as a basic tool for researchers for many decades [6]–[11]. As well as enriching our understanding of nonlinear (chaotic) dynamics, these simple models (also known as population or deterministic skeletons) have provided further insights into boom and bust behaviour in animal populations. Although there has been some success in the observation of chaos in laboratory experiments [12], [13] and in childhood diseases such as measles [14], the clinching evidence of chaos in natural populations remains elusive and this has generated considerable discussion on whether animal populations are, in general, chaotic [14]–[25].

The basis for such arguments is rooted in the finding that the pattern of fully chaotic fluctuations shown by deterministic skeletons [6]–[8] is not consistent with those observed in field data [17], [19], [20], [26], [27]. A related issue is whether the modelling of animal populations should rely only on deterministic skeletons or consist of both deterministic and stochastic elements [28], [29], while recognising the limitations imposed by the assumption of constant model parameters [30]. Motivated by such arguments, there has been a need to introduce more biological realism into mathematical models of animal populations [26], notably via the introduction of essential ecological processes (such as the migration of individuals competing for food, shelter and/or mating partners) in terms of feedbacks to population skeletons.

**Figure 1:**
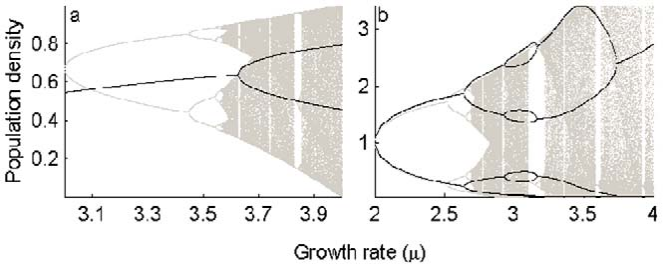
Predictive dynamics of the model 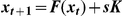. They are for functional forms (a) 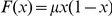 and (b) 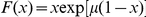. The bifurcation plots in grey are for the unperturbed maps (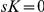), while those in black represent the perturbed maps with (a) 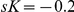 and (b) 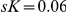.

Accounting for the migration of individuals, a class of structurally perturbed population models studied in previous work [27], [31] can be written as
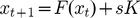
(1)where 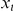 is the population size at growth generation 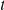. The function 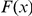 is a single-humped map (e.g. 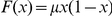) representing insect population growth with non-overlapping generations. Model dynamics are controlled by the growth parameter 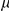 and 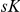 represents a perturbation to the basic model. The parameter 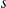 presets the direction of migration, while 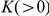 determines the amount of feedback. Under the constant-feedback formalism, deterministic chaos of population models is fully suppressed (Fig. 1). (Note that while chaos can still be found, careful examination of the 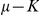 parameter space in [27] and [31] reveals that chaotic behaviour of (1) lies within an extremely small region of parameter space.) The predictive behaviour of these models, combined with accumulated insights into the theory of chaotic dynamics, led to the suggestion that perhaps chaos control techniques could help in detecting chaos in natural populations [19], [32].

Although the model is simple for suppressing chaotic dynamics, it requires *a priori* knowledge of 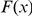 governing the underlying dynamics at any population patch [32]; *emigration* (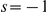) as feedback works well with 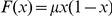 [31], while *immigration* (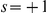) is required when 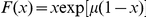 [26], [27], [33]. In retrospect, although the aim (to understand how small structural changes to population skeletons may suppress chaotic behaviour) of previous work [27], [31] was well achieved, these approaches lack the ability to be a general formulation on two counts. Firstly, there is no robust method of identifying a particular form of 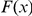 that may govern the growth dynamics of a population; trial and error experiments are required [32]. Secondly, and perhaps most importantly, if the current findings of population cycles (ranging from regular to quasicyclic to weakly chaotic or chaotic) in population data [17], [20], [34] are of relevance to understanding complexity in natural settings, then the predictive behaviour of (1) seems to be biased towards the prevailing view of asymptotic equilibrium dynamics in population ecology.

The aim of this paper is to overcome the drawbacks of the constant feedback mechanism. The suggested approach does not require *a priori* knowledge of the governing equation, perturbing population skeletons in an evolutionary way instead of requiring constant feedbacks. We observe the same repertoire of patterns, from predictive (equilibrium/periodic) states to non-chaotic aperiodic oscillations to chaotic dynamics, across different population models in agreement with the observations in animal census data. In addition, results on the existence of multiple attractors provide further insights into the importance of transient dynamics, a subject of growing research interest in ecological studies [35]-[37]. These findings suggest that such a feedback method may be applied to deterministic skeletons for a better insight into regulatory processes in natural populations.

## Materials and Methods

We present a new framework for modelling structural perturbations to the deterministic skeletons of single-species insect populations as
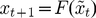
(2)where 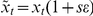 is the resident population size at generation 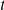. The population size at any generation depends on two parameters, namely the gain parameter 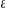, a constant quantity that moderates the magnitude of migration, and the direction parameter 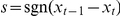 that can change through time, subjecting the patch dynamics to non-fixed (*inward* or *outward*) migration.

Because the model involves the population density from the previous generation, this is no longer a one-dimensional problem. We can instead rewrite (2) as the two-dimensional map
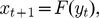
(3)

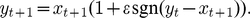
(4)


Here, the population growth phase (3) is followed by the migration phase (4), in which individuals migrate *from* or *to* a refuge. The migration process results in a net population 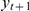 (the resident population) at the habitat at generation 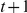, which reproduces for the next generation, and the cycle of reproduction and migration continues.

In this way, model (2) receives perturbations at generation 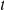 either as immigration or emigration of individuals from or to an outside refuge. Such non-fixed directional flow of migrants can be found in nature and it is well-documented [38] that species living in source-sink metapopulations can persist only when net source-to-sink, with occasional net sink-to-source, migration exists.

### Numerical Simulations, Lyapunov Exponents and Bifurcation Plots

Here, we consider the behaviour of the logistic map 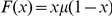, but note that the same procedures can be followed for numerical experiments with other systems such as the Ricker and Hassell maps. Individual simulations are carried out in two steps. In the first, a simulation of (2) is initialised with the initial population density selected at random from the interval (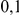) and run for more than 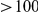 generations with 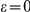. The final population density is stored and used as 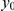, the initial resident population density. In the second step, the gain parameter 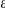 is set to a desired value (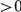) and the model is run for a number of generations, with the resident population densities recorded.

The Lyapunov exponent for the logistic map is calculated as
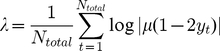
(5)where 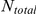 is the total number of generations (on the order of 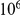). Lyapunov exponents are a measure of determining the long-term or asymptotic dynamics of nonlinear systems; negative 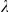 for a given growth rate 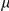 is indicative of predictive map dynamics, while positive 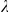 suggests chaotic population dynamics.

Bifurcation plots are obtained by plotting the resident population densities against the values of a control parameter (also known as the bifurcation parameter). Only the last 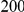 density values (after discarding transient dynamics) are recorded and plotted at selected values of the bifurcation parameter. Both the growth rate 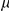 and gain parameter 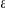 are used as the bifurcation parameter.

### Insights from the Analysis of a Simplified Version of Model (2)

Here, we present a simple analysis of (2) by imagining an idealised situation. The direction component (i.e. 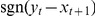) can be expressed as 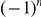 where 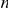 intermittently takes either the value of 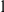 (when 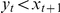) or 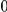 (when 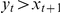). Thus, the migration phase of the population dynamics can be re-written as
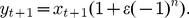
(6)


First, consider the case when 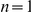, i.e. 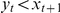, so that (2) for the logistic map can be rewritten as
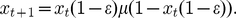
(7)


The equilibrium behaviour of (7) can be easily analysed and we find that
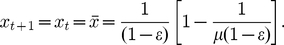
(8)


To determine the stability of the fixed point, differentiating (7) with respect to 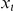 and substituting 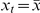 gives
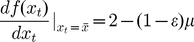
(9)


(where 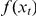 is the right-hand side of (7)), whereupon using the standard criteria for the stability of fixed points in one-dimensional maps [39], we obtain the stability condition as 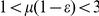. The 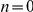 case (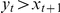) can be similarly analysed, giving the stability condition 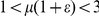. For the growth rate 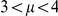, there exist non-zero 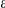 values such that the former condition (when 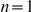) leads to the controlling of chaotic behaviour, while the latter leads to anti-control behaviour.

### Insights from a Fixed-Point Analysis of the General Model (2)

For a general function 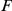 representing the population model of interest, the steady-state values 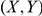 of the model (from (3) and (4)) are given by the solutions to
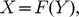


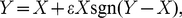
(10)or equivalently
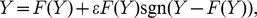
(11)leading to the two steady-states 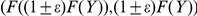, since 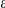 and 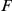 are always positive. To assess the stability of these equilibria, we calculate the Jacobian matrix 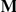 of (10), so that using the fact that 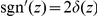 (where 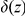 is the Dirac delta function) and, for our model, 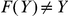, we obtain
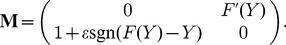
(12)


For asymptotic stability of the critical point 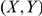, we require all eigenvalues 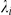 of 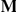 to satisfy 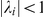 (for all 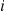). [40] demonstrate that a necessary and sufficient condition for this to hold is when
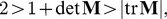
(13)which corresponds here to the requirement that
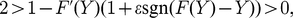
(14)so that solving both sides of the inequality leads to the condition for asymptotic stability of the steady-states as
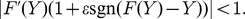
(15)


Determination and analysis of the 2-cycle solutions is outlined in Appendix S1.

#### Analysis with the logistic population model

Substituting 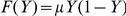 into (10) and solving gives four non-trivial steady-states (see Appendix S2), the biological relevance of which depend on the parameter regimes of interest; in particular, solution in Maple reveals the need to consider the values of 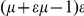 and 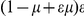. Since we are predominantly interested in chaotic regimes of the logistic map (when 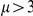) and we define 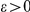 (with particular interest in small values of 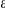), we have 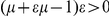 and 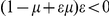, leading to the two steady-states
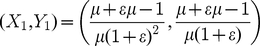
(16)and
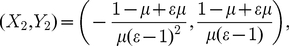
(17)both of which reduce (as expected) to the standard solution 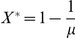 when 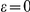. Thus, the steady-state in the unperturbed system splits into two new equilibria when 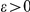 and it is readily shown that 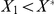 and 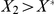.

To assess the stability of 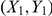, we substitute 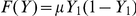 into (15), leading to the conditions on 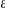 for asymptotic stability as
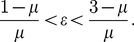
(18)


However, when 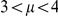, the right-hand side of (18) is never satisfied (since 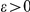) and 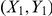 is therefore always unstable. Repeating this process with 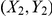 requires
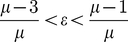
(19)for asymptotic stability of this critical point and 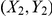 is therefore a stable steady-state when (19) holds.

#### Analysis with the exponential population model

Repeating this analysis for the exponential map with 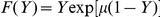 gives four non-trivial steady-states (see Appendix S2), the biological relevance of which depend on the parameters 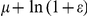 and 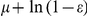. It is clear, however, that since 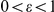, we always have 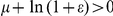 and 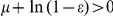, giving the two steady-states of interest
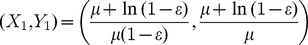
(20)and
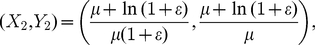
(21)both of which reduce, as expected, to the standard solution 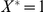 when 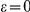. Thus, the steady-state in the unperturbed system splits into two new equilibria when 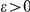 and it is readily shown that 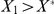 and 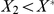.

To assess the stability of these equilibria, we substitute 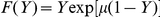 into (15), from which we find that 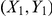 is asymptotically stable when
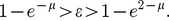
(22)


The left-hand side of (22) is always satisfied, while the right-hand side is only satisfied at relatively high values of 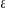 (e.g. when 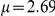 at the point where chaos just begins in the exponential map with 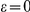, we require 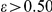 for stability of 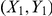). Repeating this for 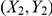 leads to the stability condition
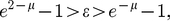
(23)the left-hand side of which is never satisfied for 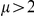, which incorporates the region where chaos occurs and where we are interested, and thus 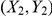 is never stable.

## Results

### Numerical Verification of Fixed-Point Behaviour

**Figure 2:**
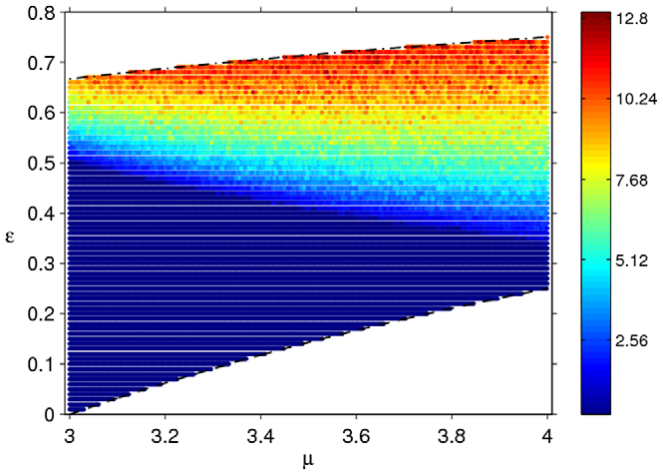
Coexistence of the non-trivial fixed-point with the trivial one (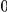). The colour bar (in 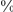) shows the extent to which we expect the occurrence of this behaviour as the strength of the gain parameter 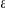 increases at a given 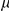. The lower dashed line was derived from the lower bound on 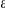 from the equation (19), while the dot-dash line derived from the upper bound.

Within the bounds of 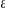 given by (19), we expect the model to have a single basin of attraction towards the equilibrium point 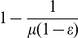. Note the lower bound on 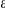 is the same minimum value for the stability of the fixed point derived from the analysis of the 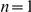 case earlier; it provides a cross-check on both approaches. However, when the model is simulated with different initial population densities, trajectories are attracted to both stable fixed points – the non-trivial equilibrium and zero (Fig. 2). This is not surprising though; in the derivation of our analytical results, we assume that the asymptotic dynamics are independent of the initial conditions. While this may be the case for the majority of nonlinear dynamical systems, for systems like (2) where there is a possibility that the dynamics may occasionally get pushed into other regimes, this assumption may not always hold.

### General Observations

**Figure 3:**
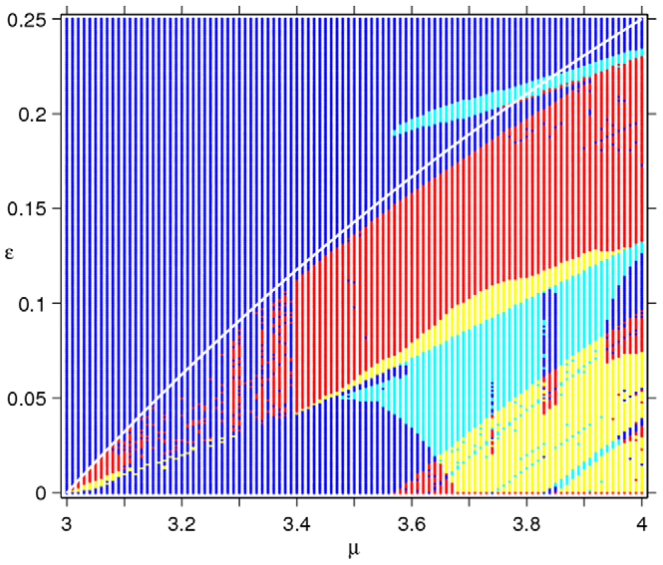
The 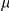
–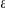 parameter space of the dynamical patterns of model (2). Here, 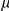 is incremented with a stepsize of 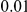, while a stepsize of 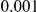 is used for 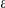. The grid points coded with blue denote predictive behaviours (periodic or fixed point) – the dynamics independent of initial population densities. Red denotes chaotic behaviour, yellow NAO behaviour and cyan where model dynamics eventually settle down to one of the coexisting attractors. Colour coding in the parameter space is carried out on the basis of the model's long-term dynamics (removing transients for 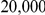 generations and using the remaining 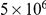 generations for the calculation of 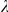). The white line represents the analytical result of the minimum value of 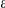. In the region above this line, the model is expected to have stable fixed-point behaviour.

**Figure 4:**
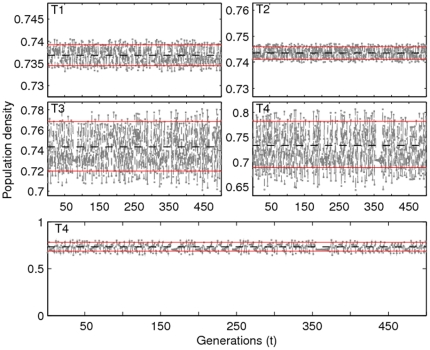
Changing patterns of long-term dynamics. Here, the bifurcation plots are obtained by plotting the resident population densities from the last 200 generations after discarding transient dynamics. Both bifurcation parameters 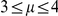 (left) and 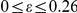 (right) are incremented with a stepsize of 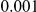. The left-panel plots are for different values of the gain parameter 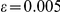 (a1), 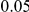 (a2), 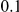 (a3) and 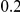 (a4). The right-panel plots are for 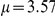 (b1), 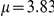 (b2), 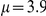 (b3) and 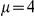 (b4).

Though we have verified our findings for different forms of 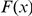, results are given for the logistic map. Firstly, within the limiting value (the lower bound from condition (19)) of the gain parameter 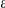, there is no feedback-induced escape to -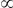 as observed in [31] (which occurs due to violation of the necessary condition 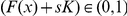 for avoiding escape). This conclusion results from the extensive numerical model runs required to construct the parameter space in Fig. 3. Secondly, unlike previous results [27], [31], the dynamics of (2) are of predictive or chaotic type across values of the gain parameter within this limiting bound (Fig. 4).

**Figure 5:**
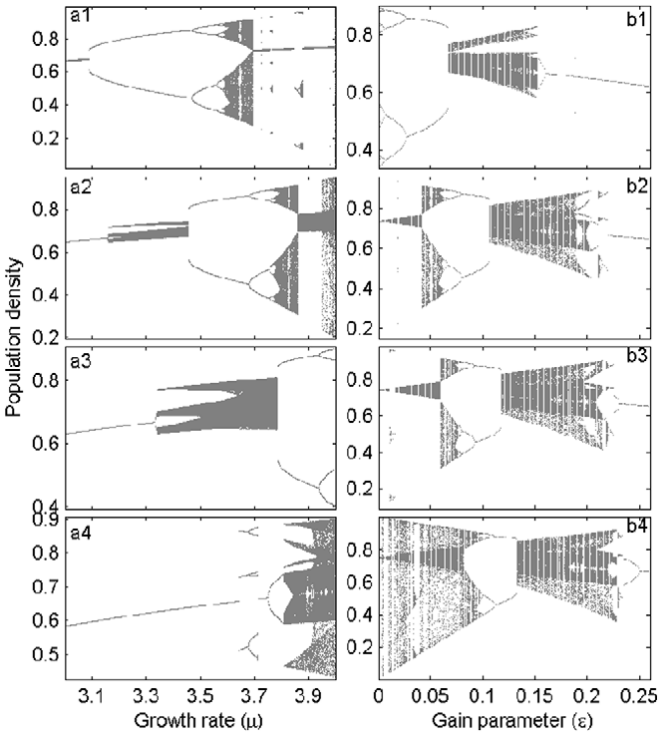
Illustration of Non-chaotic Aperiodic Oscillations (NAO). Time series are for different combinations of the gain parameter 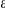 and growth rate 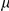: (T1) 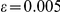, 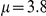; (T2) 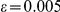, 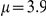; (T3) 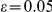, 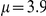 and (T4) 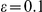, 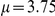. Only 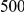 generations are used in all four plots after discarding transients. Two horizontal lines are given by 
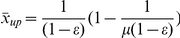
 and 
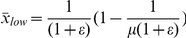
. The two values are derived from the analysis of the simplified version of (2) for 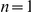 and 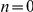, respectively. The time series T4 is plotted on a different *y*–scale to emphasize small fluctuations in the time series. The dashed-line represents the unstable fixed point of the map with 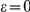.

An important feature of the model dynamics is the occurrence of non-chaotic aperiodic oscillations (NAO). When analysed on a suitable scale, NAO behaviour looks like a noisy limit cycle (Fig. 5), with population densities fluctuating around the (unstable) fixed point 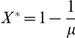. One diagnostic feature of this behaviour is that population trajectories spend more than 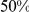 of their time in the region bounded by the two population densities (shown by solid lines in the figure plots).

### Parameter Space of the Model Dynamics

Long-term behaviour of the model, summarised on a 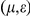 parameter grid in Fig. 3, presents a complex mosaic. The dynamics at each grid point are characterised by Lyapunov exponents for a set of 100 initial population densities such that 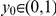. If the condition 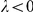 is satisfied for all simulations at a 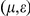 grid point, the model behaviour is termed predictive (fixed-point or periodic) and such grid point is coloured blue. For NAO dynamics at the grid points displayed in yellow, we find that all 100 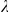 values are very close to 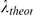 where 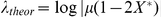. NAO behaviour is cross-checked using the other diagnostic measure discussed above. Any other behaviours of 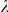 are found to display chaotic dynamics and the corresponding grid points are displayed in red. The cyan region (discussed further below) differs significantly from the rest in that model dynamics are found to display coexisting attractors. As an aside, we note that use of a more analytical technique (such as one based on mathematical continuation) to demarcate boundaries of different dynamical patterns in the parameter space is non-trivial because of the coexistence of attractors.

### The Changing Pattern of Long-Term Dynamics

The pattern of long-term dynamics (Fig. 4) is observed to change with variation in the control parameters, but not in any particular order. Strong chaotic fluctuations of the unperturbed logistic map (cf. the 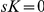 plot in Fig. 1a) are changed to mostly NAO dynamics when 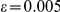 (Fig. 4a1), through to mildly chaotic plus NAO for 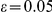 (Fig. 4a2) and dynamics constituted by a combination of NAO and periodic behaviour when 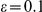 (Fig. 4a3). Further increase in the gain parameter to 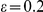 is found to completely suppress chaos at 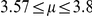, while maintaining weak chaotic fluctuations for higher growth rates (Fig. 4a4).

Plots with 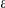 as a control parameter reveal (Figs. 4b1 to 4b4) the phenomenon of period doubling reversal (PDR) in model dynamics for weakly, as well as strongly, chaotic growth rate. This is more conspicuous at 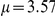. The PDR phenomenon (Fig. 1b), reported in [27], differs from our observations in that the PDR regime here is interspersed with NAO or chaotic cycles. Note that variability in the population dynamics disappears as 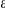 approaches its lower bound of 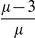 derived from the fixed-point stability analysis. Periodic or fixed-point dynamics at the limiting value of 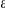 is confirmation that this feedback acts to control chaos. However, as noted above in the case of 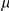 as the bifurcation parameter, the progression (that is, the appearance of dynamic patterns as 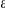 increases) towards suppressing chaos at different 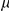 values is not same (see Figs. 4b1 to 4b4 in conjunction with the parameter space below the white line in Fig. 3).

### Coexistence of Attractors

**Figure 6:**
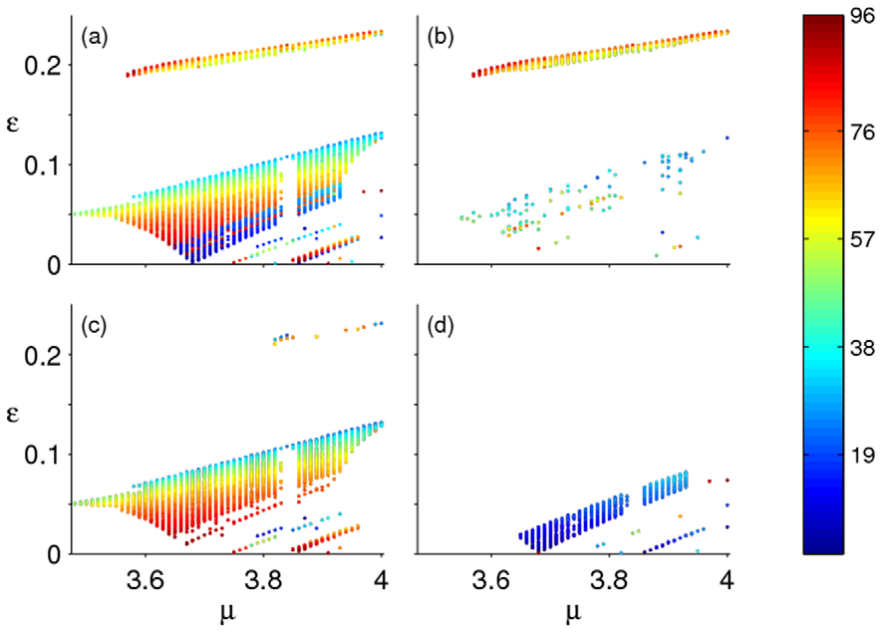
Coexistence of attractors. Plot (a) shows the extent to which one attractor, for any values of 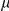 and 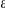, can coexist with another attractor (expressed as a percentage on the colour bar). The other plots are (b) one periodic attractor coexisting with either another periodic attractor or an NAO attractor, (c) a periodic attractor coexisting with an NAO attractor only and (d) an NAO attractor coexisting with a chaotic attractor.

The cyan area of parameter space has two distinct regions, with a virtual line at 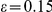 acting as a separator (Fig. 6a). In this region, numerical analysis shows that coexisting attractors occur in one of these combinations: (i) all are periodic attractors; (ii) periodic attractors coexist with the NAO attractor; or (iii) the NAO attractor coexists with the chaotic attractor. However, when we examine the dynamics on a majority basis, we can identify three regimes of note. First, for some values of 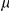 and 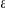 (Fig. 6b), the main attractor is a periodic attractor that coexists either with another periodic attractor or with the NAO attractor. We find that the coexisting periodic attractors are of even period, with one being a multiple of the other. Occasionally, however, we observe an odd-period attractor coexisting with an even-period one (such as the coexistence of period-3 and period-2 attractors). Second, the main NAO attractor can coexist with the periodic attractors (Fig. 6c) and this coexistence has the largest domain. Here, for any value of 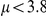, the gain parameter 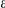 needs to be less than 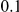 for coexistence to occur. Third, the NAO attractor is found to coexist with the chaotic attractor (Fig. 6d). We find that the attraction of temporally evolving population trajectories towards one of the competing attractors depends on the initial population densities. Such dependence on initial densities has been reported to be abundantly present in natural populations as a result of the impact of environmental noise on population dynamics [34]. This echoes well the observations made elsewhere [11] on the importance of the historical origins on outcomes of dynamical systems, though contrasts with other studies [27], [31] where the behaviour of perturbed models is found to remain almost always of the periodic type – insensitive to the initial conditions.

#### Illusion of the coexistence of attractors

**Figure 7:**
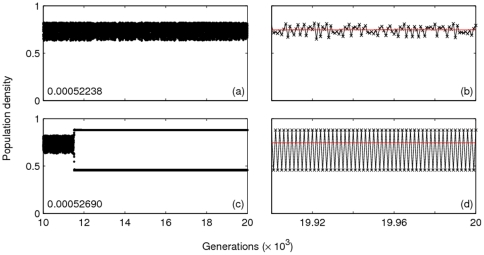
Effect of transients of varying length. Population densities for 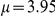 and 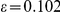, plotted as points, are shown for the last 10,000 generations. Initial population densities are shown in the bottom left corners of (a) and (c). The last 100 generations of these time series are plotted as line plots in (b) and (d) to clearly contrast the NAO behaviour in (a) with the final periodic behaviour in (c).

**Figure 8:**
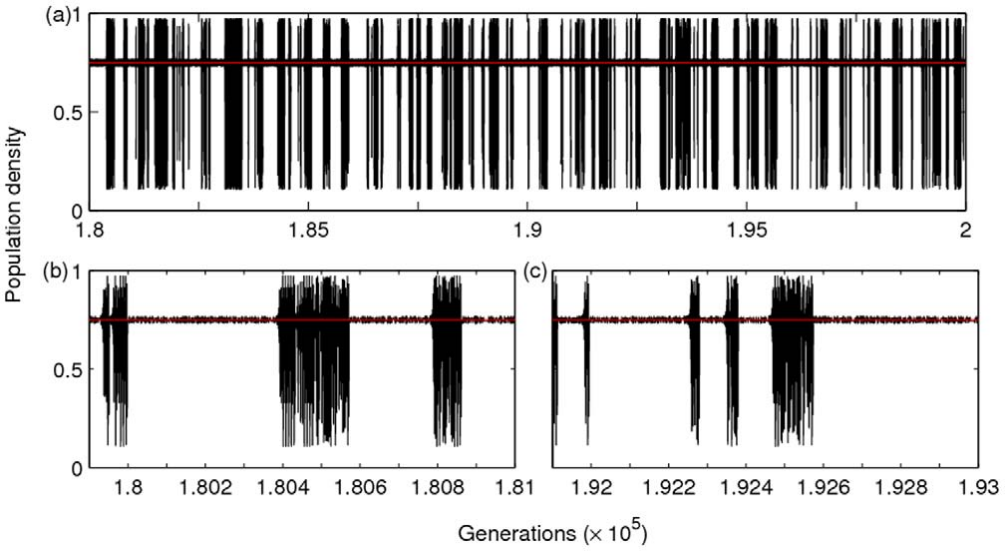
Effect of intermittent transient dynamics. The model was run for a total of 2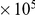 generations, with 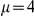 and 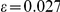. The time series in (a) shows population densities for the last 20,000 generations. The time series in (b) and (c) have been plotted on a shorter time-scale to zoom in on the duration of the NAO windows. Note that ticks on the *x*-axis in the bottom plots are separated by 100 generations.

Apart from the coexistence of attractors discussed above, long transient dynamics are found to impact judgement on the appearance of possible coexistence of attractors. Two examples are shown for 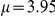 and 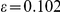 (Fig. 7) and 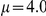 and 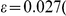 Fig. 8). The long-term dynamics for each of these parameter combinations is a single attractor, but the nature of the population trajectories appears to suggest the coexistence of two attractors. Of course, as explained below, this illusion of coexisting attractors critically depends on the timing and frequency at which we sample or record the population trajectories.

The mechanisms behind such behaviour are of two types. In the former case, transient dynamics of variable duration are responsible for the illusion of this coexistence. For example, the time series in Fig. 7 are generated by running the model with almost identical initial population densities. However, as shown in Figs. 7a & 7b, the population trajectory mildly fluctuates around the unstable equilibrium 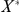 (shown by the horizontal line) until the end of the 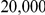
th growth generation, while the predictive time series (shown in Figs. 7c & 7d) settles down on the periodic attractor by the end of the 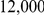
th generation (Fig. 7c).

In the latter case, changing dynamical behaviour from one type (in a temporal window) to another in the next temporal window may create the illusion of coexistence. For example, in Fig. 8 the final attractor is an NAO attractor intermittently interrupted with chaotic dynamics. However, when examined more closely, several windows of the NAO dynamics of more than 200 generations long are present in both Figs. 8b and 8c. Sampling or recording of the population trajectories at certain periodicity can generate the illusion that the dynamics are changing from one attractor to another. This intermittent behaviour is not limited to the model under discussion; it has also been observed in the dynamics of forced SEIR (susceptible, exposed, infectious and recovered) models of childhood diseases (e.g. measles outbreaks; see Fig. 8 in [41]). This behaviour reportedly occurs due to ‘transient’ periodicity, a natural part of (deterministic) chaotic dynamics, which has been argued to be responsible for biennial episodes in measles data [41].

## Discussion

The approach presented does not require trial and error experiments to select the most appropriate structural perturbation to a given population model, thereby providing a uniform mechanism. We observe the same repertoire of patterns, from equilibrium to NAO to chaotic behaviour across different population models. NAO behaviour can be compared with quasicyclic (almost periodic, but with varying amplitude) fluctuations observed in natural populations. Such complex dynamics have been reported to be rife as a result of the interplay between stochastic noise and the non-linearity present in population growth processes [34] and are thought to be one of the factors responsible for obscuring the presence of chaos in natural populations. To the best of our knowledge, this behaviour has not been reported before in structurally perturbed population models. However, such trajectories are not limited to models of insect populations; they have also been observed in the dynamics of the seasonally-forced SEIR epidemic model [41].

Our results suggest that caution should be exercised when interpreting empirical data of animal populations. Chaotic/wild fluctuations in data may appear due to intrinsic-extrinsic feedback (accounting for some aspect(s) of demographic structure), even for parameter values which, in the absence of such feedback, can only cause equilibrium dynamics (cf. the red region of Fig. 3 for 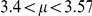). The same argument also appears to be valid for the opposite case (cf. the blue region of Fig. 3 for 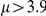 and 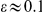). While it is not possible to draw a greater parallel between our results and the observations of [42] due to the different level of model complexity involved, the deterministic skeleton of rodent population dynamics in the absence of structural perturbations (namely not accounting for rainfall effects) can only produce equilibrium dynamics (see [42], pp 486). When rainfall patterns are taken into account, the model is able to reproduce strong variability in time series data.

Furthermore, our model displays the existence of competing attractors. Coexistence of multiple attractors has been reported in the dynamics of discrete stage-structured competition models ([43] and references therein) and those of mechanistic infectious disease models ([44], pp. 155-189), where initial population densities are found to be responsible for the emergence of coexisting attractors. Stage-structured discrete models are by far more complex than the model system here. The presence of multiple attractors in a simple structurally perturbed model like the one here suggests that perhaps the coexistence of attractors is rule rather than an exception in natural populations.

In addition, we find that, for some values of model parameters for which the asymptotic dynamics are a single periodic attractor, transients of variable length could suggest that the population dynamics have more than one attractor. Initial population densities are again found to play a key role in shaping population trajectories. The case where population dynamics continuously appear to be transient, such as when NAO behaviour is interrupted by chaotic fluctuations (Fig. 8), may also mislead us in believing that the population dynamics hop between two attractors (depending on the sampling frequency at which animal censuses are carried out). In recent years, transient dynamics have been argued to play a more critical role in shaping ecological populations than asymptotic equilibrium dynamics [11], [28], [35], [36]. Understanding new mechanisms by which transients can impact our judgement is therefore a clear necessity, as well as a priority for better conservation and sustainable management efforts targeted at natural populations.

## Supporting Information

Appendix S1 **2-cycle determination from analysis of the general model (2).**
(DOC)

Appendix S2 **Equilibrium solutions of the logistic and exponential maps.**
(DOC)
