# Correction: Structural Perturbations to Population Skeletons: Transient Dynamics, Coexistence of Attractors and the Rarity of Chaos

**DOI:** 10.1371/journal.pone.0157617

**Published:** 2016-06-10

**Authors:** Brajendra K. Singh, Paul E. Parham, Chin-Kun Hu

The images for Figs 4 and 5 are incorrectly switched. The image that appears as Fig 4 should be Fig 5, and the image that appears as Fig 5 should be Fig 4. The figure captions appear in the correct order.

**Fig 4:**
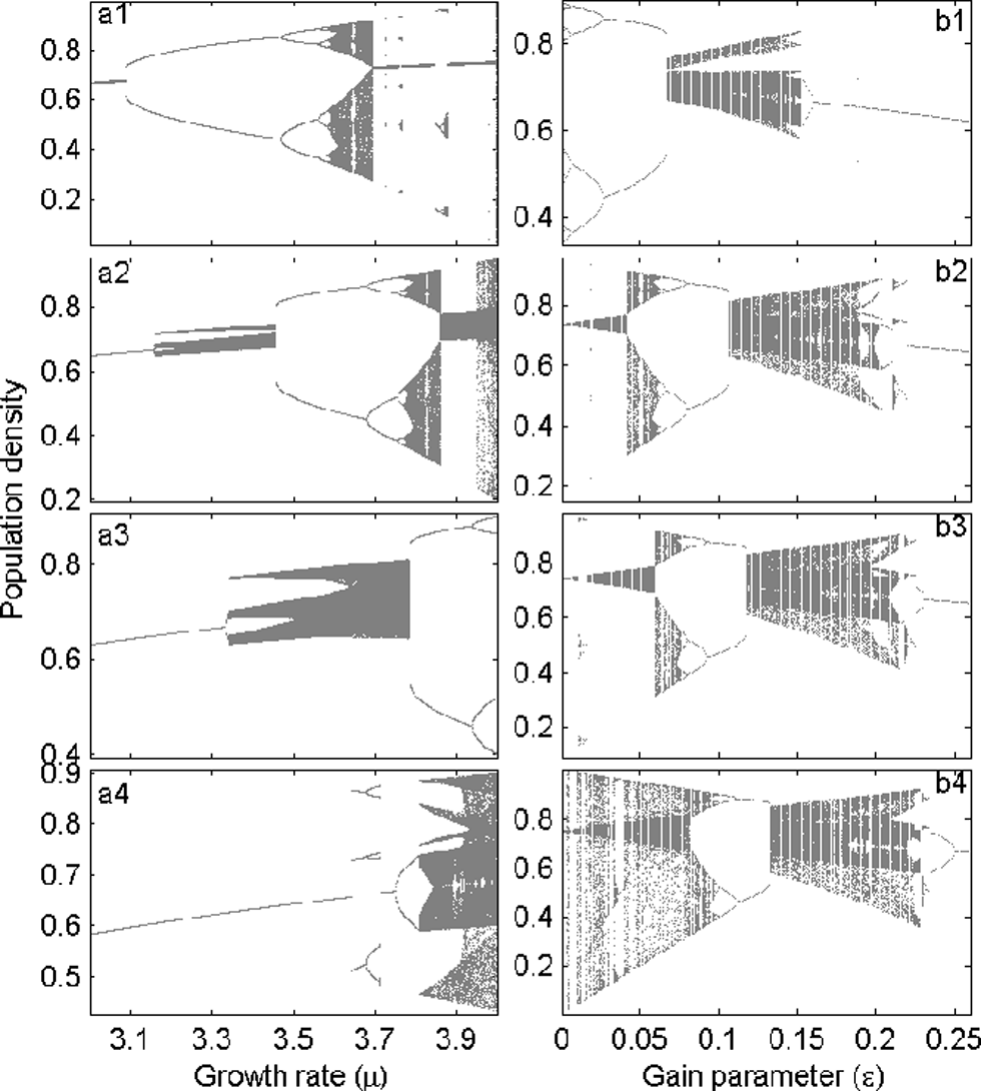
Changing patterns of long-term dynamics. Here, the bifurcation plots are obtained by plotting the resident population densities from the last 200 generations after discarding transient dynamics. Both bifurcation parameters 3≤*μ*≤ 4 (left) and 0≤*ε*≤0.26 (right) are incremented with a stepsize of 0.001. The left-panel plots are for different values of the gain parameter *ε* = 0.005 (a1), 0.05 (a2), 0.1 (a3) and 0.2 (a4). The right-panel plots are for *μ* = 3.57 (b1), *μ* = 3.83 (b2), *μ* = 3.9 (b3) and *μ* = 4 (b4).

**Fig 5:**
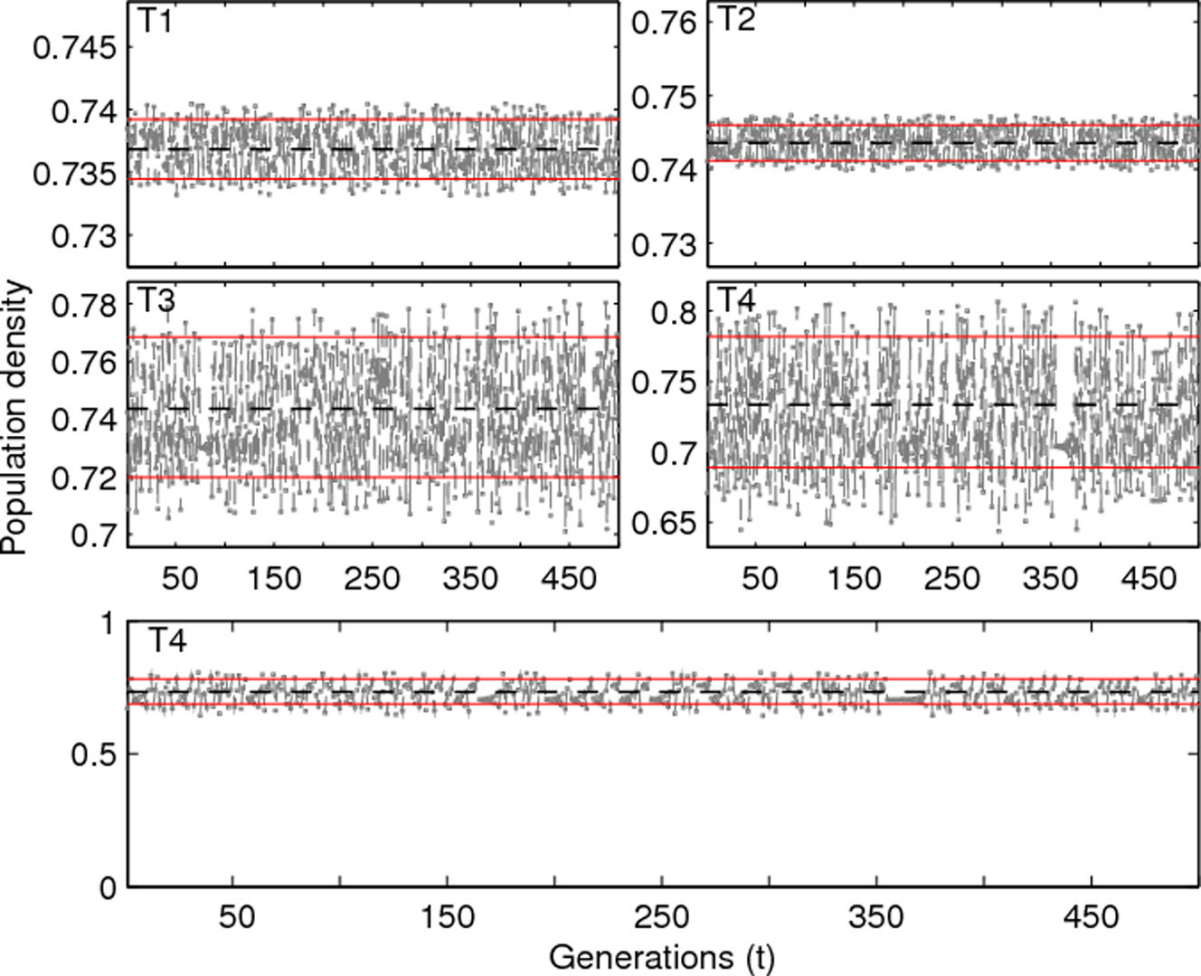
Illustration of Non-chaotic Aperiodic Oscillations (NAO). Time series are for different combinations of the gain parameter *ε* and growth rate *μ*: (T1) *ε* = 0.005, *μ* = 3.8; (T2) *ε* = 0.005, *μ* = 3.9; (T3) *ε* = 0.05, *μ* = 3.9 and (T4) *ε* = 0.1, *μ* = 3.75. Only 500 generations are used in all four plots after discarding transients. Two horizontal lines are given by ${\bar{x}}_{up}=\frac{1}{\left(1-\varepsilon \right)}(1-\frac{1}{\mu (1-\varepsilon )})$ and ${\bar{x}}_{low}=\frac{1}{\left(1+\varepsilon \right)}(1-\frac{1}{\mu (1+\varepsilon )})$. The two values are derived from the analysis of the simplified version of (2) for *n* = 1 and *n* = 0, respectively. The time series T4 is plotted on a different y–scale to emphasize small fluctuations in the time series. The dashed-line represents the unstable fixed point of the map with *ε* = 0.
