# Supplementary material for: Structural Perturbations to Population Skeletons: Transient Dynamics, Coexistence of Attractors and the Rarity of Chaos
Source: PLoS One. 2011 Sep 19;6(9):e24200. doi: 10.1371/journal.pone.0024200 (PMC3176270; doi:10.1371/journal.pone.0024200)
Supplement: Appendix S1 — 2-cycle determination from analysis of the general model (2). (DOC) [file pone.0024200.s001.doc]

**Appendix S1**

**2-cycle determination from analysis of the general model (2)**

In the general model, the 2-cycles may be calculated from the solutions to

(24)

with the general 2D map preserving area when $|\text{det}\mathbf{M}|=1$, a property arising for Poincaré return maps through Hamiltonian systems [45].

For the logistic map, the 2-cycles may be determined by substituting $F(Y)=\mu Y(1-Y)$ into (24) and solving the resultant (order-13) polynomial. Solutions of this polynomial consist of the trivial solution, the four steady-states discussed in the main text and eight other solutions that correspond to four 2-cycles, one arising for each of the fixed points. Thus, $(X_{2},Y_{2})$ (the steady-state of interest) undergoes a period-doubling bifurcation when and although the limits of this 2-cycle may be expressed analytically in closed-form, these solutions (plus their stability criteria) are too unwieldy to reproduce here. Note also that for $(X_{2},Y_{2})$, the system becomes an area-preserving map when increases such that $(X_{2},Y_{2})$ becomes stable, as well as representing the point at which this steady-state undergoes a period-doubling bifurcation.

The 2-cycles for the exponential map may be determined in the same way, namely by substituting $F(Y)=Ye^{r(1-Y)}$ into (24) and solving the resultant polynomial, but this is considerably more difficult for the exponential map due to its functional form and we therefore do not analytically pursue this any further here. Note also that for $(X_{1},Y_{1})$, the system becomes an area-preserving map when and , while this is never possible for $(X_{2},Y_{2})$.
