# Supplementary material for: Structural Perturbations to Population Skeletons: Transient Dynamics, Coexistence of Attractors and the Rarity of Chaos
Source: PLoS One. 2011 Sep 19;6(9):e24200. doi: 10.1371/journal.pone.0024200 (PMC3176270; doi:10.1371/journal.pone.0024200)
Supplement: Appendix S2 — Equilibrium solutions of the logistic and exponential maps. (DOC) [file pone.0024200.s002.doc]

**Appendix S2**

**Equilibrium solutions of the logistic and exponential maps**

To determine equilibrium solutions of the logistic population model

$$X=\mu Y(1-Y),$$

(25)

solution in Maple for the non-trivial states gives the $(X,Y)$ equilibria

• if and ,

• and if and ,

• if and .

These solutions are therefore dependent on two parameter regimes. Since we are only considering chaotic regimes (so $3<\mu<4$) and we define , we have and we can therefore ignore the solution in the third bullet point. In addition, since we are only considering low values of (up to around 0.25), we can show that for $3<\mu<4$ (since we would otherwise be requiring when $\mu=3$ up to when $\mu=4$) and hence we can also ignore the solution in the first bullet point. This leaves the two solutions in the second bullet point and we thus define

(26)

and

(27)

both of which reduce, as expected, to the standard solution $X^{*}=1-\frac{1}{\mu}$ when .

Repeating this analysis to determine the equilibrium solutions of the exponential population model

$$X=Y\text{exp}[\mu(1-Y)],$$

(28)

and solving in Maple for the non-trivial states gives the $(X,Y)$ equilibria

• if and ,

• and if and

• if and .

As before, the existence of these solutions in the regimes of interest depends on two threshold quantities. It is clear that since , we always have and we can thus ignore the solution in the first bullet point. In addition, since , and we can also ignore the solution in the third bullet point, leaving the steady-states in the second bullet point and we label these as

(29)

and

(30)

both of which reduce, as expected, to the standard solution $X^{*}=1$ when .
